# *TAS2R38* Bitter Taste Receptor Expression in Chronic Rhinosinusitis with Nasal Polyps: New Data on Polypoid Tissue

**DOI:** 10.3390/ijms23137345

**Published:** 2022-07-01

**Authors:** Joanna Jeruzal-Świątecka, Edyta Borkowska, Mateusz Łaszczych, Zuzanna Nowicka, Wioletta Pietruszewska

**Affiliations:** 1 Department of Otolaryngology, Head and Neck Oncology, Medical University of Lodz, 90-419 Lodz, Poland; wioletta.pietruszewska@umed.lodz.pl; 2 Department of Clinical Genetics, Medical University of Lodz, 90-419 Lodz, Poland; edyta.borkowska@umed.lodz.pl; 3 Department of Biostatistics and Translational Medicine, Medical University of Lodz, 90-419 Lodz, Poland; mateusz.laszczych@stud.umed.lodz.pl (M.Ł.); zuzanna.nowicka@umed.lodz.pl (Z.N.)

**Keywords:** bitter taste receptors, innate immunity, biomarker, sinonasal epithelial cells, polypoid tissue, taste, chronic rhinosinusitis, genetic background, asthma, *TAS2R38*

## Abstract

Studies have shown differences in *TAS2R38* receptor expression in patients with chronic rhinosinusitis (CRS) compared to healthy controls. Known agonists of *TAS2R38* stimulate epithelial cells, leading to robust intracellular nitric oxide (NO) production, which damages bacterial membranes, enzymes, and DNA, but also increases ciliary beat frequency. In this study we examined, using qRT-PCR, the expression of *TAS2R38* receptor in nasal polyps (NP) of patients with CRS (N = 107) and in inferior turbinate mucosa (ITM) of patients with CRS and controls (N = 39), and confronted it with clinical features and the severity of the disease. The expression was shown in 43 (50.00%) samples of ITM in the study group (N = 107), in 28 (71.79%) in the control group (N = 39) (*p* = 0.037), and in 43 (46.24%) of NP. There were no differences in levels of the expression in all analyzed tissues. Patients who rated their symptoms at 0–3 showed higher *TAS2R38* expression in ITM in comparison to the patients with 8–10 points on the VAS scale (*p* = 0.020). A noticeable, however not significant, correlation between the *TAS2R38* expression in ITM and the Lund–Mackay CT score was shown (*p* = 0.068; R = −0.28). Patients with coexisting asthma had significantly higher receptor expression in the NP (*p* = 0.012). Our study is the first to confirm the presence of the *TAS2R38* receptor in NP. Expression of the *TAS2R38* receptor is reduced in the sinonasal mucosa in patients with more advanced CRS with NP.

## 1. Introduction

Scientists have been looking very closely at the bitter taste receptors (*T2Rs* or *TAS2Rs*) over the past decade. These receptors became the focus of their interest mainly due to their presence in many healthy and pathological tissues of the human body. The presence of *TAS2Rs* has been proven not only in the oral cavity, but also in upper and lower respiratory system cells, thyroid, skin, gastrointestinal truck, immune system cells, heart, mammary and myometrial cells, semen and testicular cells, and even brain [1,2,3,4,5,6,7,8,9,10,11,12,13,14,15,16,17,18,19,20,21,22,23,24,25,26]. The presence of *TAS2Rs* in many neoplastic cells has also been studied. Jaggupalli et al. studied breast cancer cells and stated that the expression of *TAS2R14* and *TAS2R20 (49)* is much higher in breast cancer cells than in normal mammary tissue [2]. Stern et al. reported expression and functionality of the bitter receptor *TAS2R10* in both human pancreatic ductal adenocarcinoma (PDAC) tissue and PDAC-derived cell lines [27]. In a study of Carrai et al., patients with nonfunctional alleles of *TAS2R38* receptors had an increased risk of colorectal cancer [28]. Finally, it was established that single nucleotide polymorphisms (SNPs) in the *TAS2R38* gene may contribute to individual differences in susceptibility to respiratory infections—in particular, to chronic rhinosinusitis (CRS) [29,30].

CRS is divided into two specific disease entities: chronic rhinosinusitis with (CRSwNP) and without nasal polyps (CRSsNP), and it is characterized by the presence of polypous lesions in the nasal cavities and/or paranasal sinuses. The role of allergy, bacterial, fungal and viral infection, congenital immunodeficiencies, ciliary impairments, bacteria forming biofilms, environmental factors, and genetic factors are emphasized in the etiopathogenesis of CRS [31,32]. Research concerning bitter taste receptors is another path in finding the causes of the disease and individual differences in its course. After the confirmation of the protective role of the *TAS2R38* receptor located in the sinonasal epithelial cells, studies were undertaken to indicate its role in individual susceptibility to upper respiratory infections [33]. Studies have shown differences in *TAS2R38* receptor expression levels in sinus mucosa of patients with CRS compared to healthy controls, as well as differences between CRS with and without nasal polyps [34].

**Figure 1:**
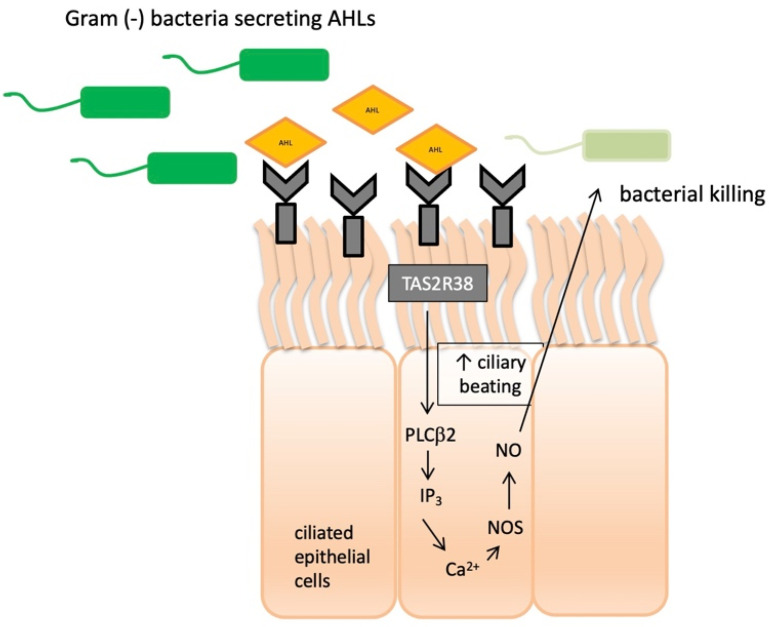
*TAS2R38* bitter taste receptor regulation in human sinonasal epithelial innate immunity. AHLs = acyl-homoserine lactones; Ca^2+^ = calcium ion; NO = nitric oxide; NOS = nitric oxide synthase; PLCβ2 = phospholipase; IP_3_ = inositol trisphosphate.

The cascade of receptor responses in the nasal epithelium plays a crucial role in regulation of innate immunity. When epithelial cells are stimulated with known agonists of *TAS2R38,* they exhibit low-level calcium responses that activate nitric oxide (NO) synthase (NOS), leading to robust intracellular NO production [35,36]. NO and its derivatives exert detrimental effects on bacterial membranes, enzymes, and DNA. Another mechanism of their action is by increasing the ciliary beat frequency through the activation of guanylyl cyclase and protein kinase G, which are phosphorylate ciliary proteins that accelerate mucociliary clearance (Figure 1). Two major *Pseudomonas aeruginosa* acyl-homoserine lactones (AHLs) were identified as agonists of *TAS2R38* [33,37,38]. It was demonstrated that *TAS2R38* detects physiological concentrations of AHLs, resulting in the activation of calcium-dependent NO production [39]. Many Gram-negative species secrete AHLs, and *TAS2R38* is thus likely to function in airway ciliated cells as a sentinel receptor for invading Gram-negative bacteria that are capable of triggering a critical defensive bactericidal response.

Taking all this data under consideration, we hypothesized that the *TAS2R38* bitter taste receptors might also be expressed in the pathological tissue of nasal polyps. We wanted to study this issue more carefully by analyzing the expression of the receptor with clinical features and the severity of the disease.

## 2. Results

The research group included 146 patients, with 107 (63 M/44 F) in the study group and 39 (25 M/14 F) in the control group. More than 60% of patients from both groups felt the bitter taste of phenylthiocarbamide (PTC). A significant majority of both groups admitted to seasonal or year-round allergies (CG: 38/36; SG: 84/78). The median SNOT-22 score in the study group was 32 (27–37), the average score in Lund–Mackey was 9 (6–14), and more than half of the patients marked 0–3 on the VAS scale (51.40%) and had scored 0–2 in Lund–Kennedy scale (55.14%) in endoscopic examination (Table 1).

Previously, different reference genes have been used for qRT-PCR while studying bitter taste receptors expression, with glyceraldehyde 3-phosphate dehydrogenase gene (*GAPDH*) being the most used [40,41,42,43]. We evaluated expression of 18S ribosomal RNA *(18S RNA)*, *β-actin*, and *GAPDH* and found that *β-actin* was the best normalizer, taking into account the coefficient of variation (CoV) of CT values, difference in expression between polyp and ITM, and the number of samples with non-zero expression (Table 2). Therefore, the expression was normalized to *β-actin*.

A total of 93 samples of polypoid tissue and 115 samples of inferior turbinate mucosa were analyzed for the *TAS2R38* receptor expression. The expression was shown in 43 (50.00%) samples of inferior turbinate mucosa in the study group and in 28 (71.79%) in the control group (*p* = 0.037). We also confirmed the expression in 43 (46.24%) samples of polypoid tissue. In 15 patients (20.55%) from the study group, the expression of *TAS2R38* was confirmed in both polyp and inferior turbinate mucosa. The levels of the expression in all analyzed tissues were similar (Table 3).

**Figure 2:**
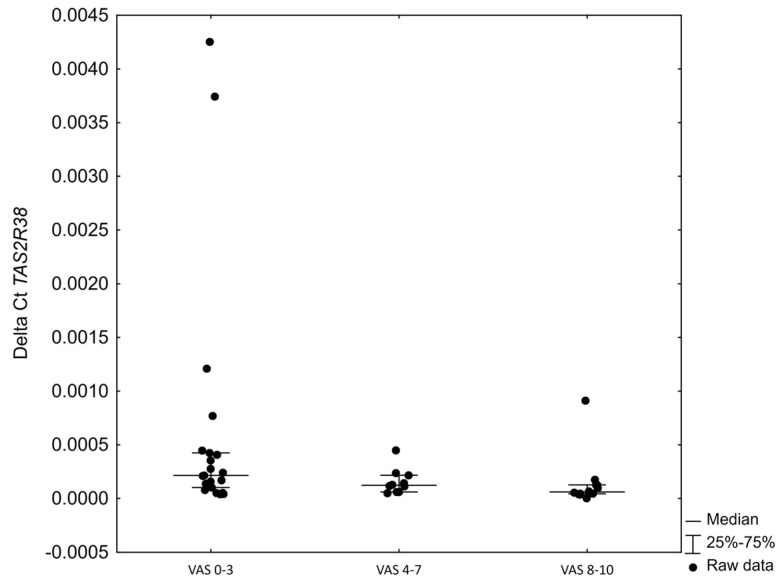
Level of TAS2R38 receptor expression in inferior turbinate mucosa within patient subgroups divided according to VAS scale ranges. Kruskal–Wallis’s analysis of variance *p* = 0.020; post hoc Dunn’s test *p* = 0.0158 between VAS 0–3 and VAS 8–10.

No correlation was found between the level of *TAS2R38* receptor expression in the polyp tissue or inferior turbinate tissue and the patient’s score on the SNOT-22 scale (*p* = 0.103 R = 0.25; *p* = 0.291 R = −0.16, respectively). Patients who rated their symptoms from 0 to 3 points on the VAS scale showed higher expression of the receptor in inferior turbinate mucosa in comparison to the patients with 8–10 points on the VAS scale (Figure 2).

**Figure 3:**
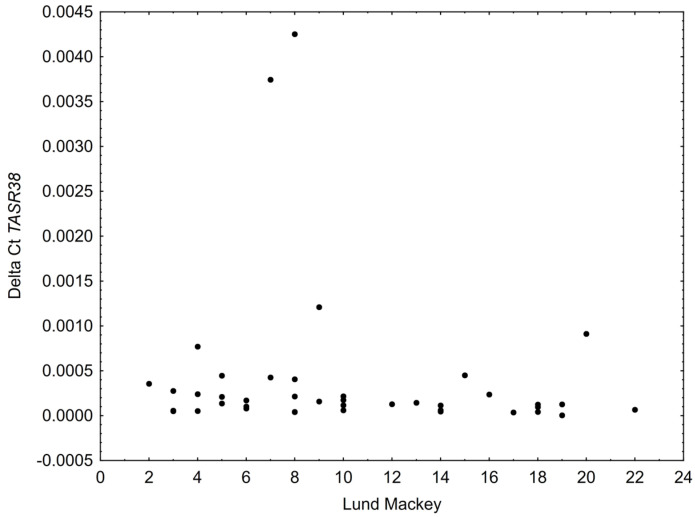
Relation between level of TAS2R38 expression in inferior turbinate mucosa and Lund–Mackay score. Spearman R = −0.28; *p* = 0.068.

No correlation was found between the level of *TAS2R38* receptor expression in the polyp tissue and the patient’s score in the Lund–Mackay scale; however, there was a noticeable relationship between the level of *TAS2R38* expression in inferior turbinate mucosa and the Lund–Mackay score, suggesting a negative correlation (*p* = 0.068; R = −0.28), as shown on Figure 3.

**Figure 4:**
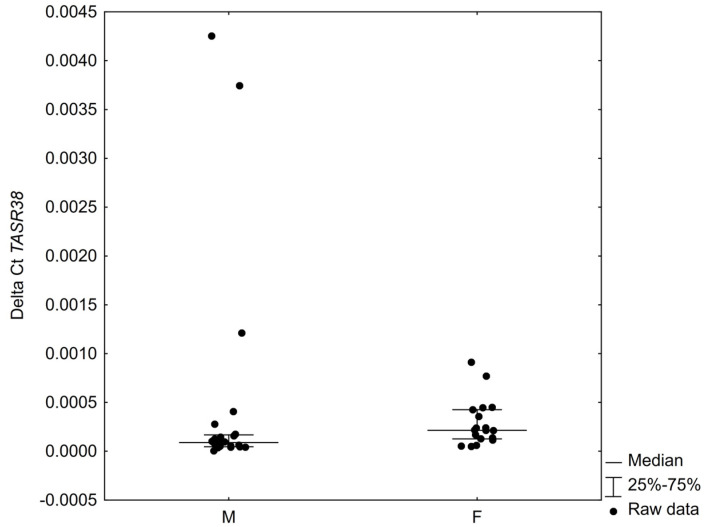
Difference between the sexes in TAS2R38 expression level in inferior turbinate mucosa *p* = 0.0195.

A statistically significant difference in the expression of the receptor in the tissue of the inferior turbinate was demonstrated between the sexes in the study group, where males had significantly lower TAS2R38 expression than females (*p* = 0.0195) (Figure 4).

**Figure 5:**
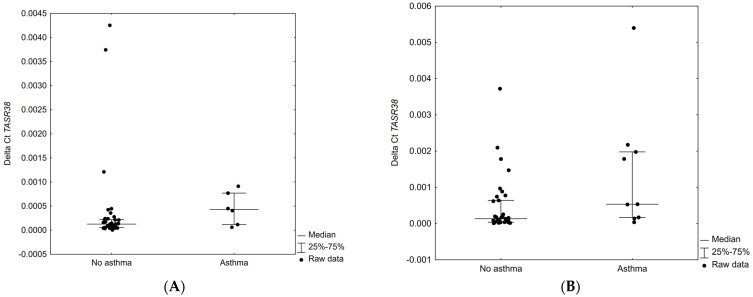
Difference between groups divided according to asthma state in *TAS2R38* expression level in inferior turbinate mucosa (**A**) and polyp tissue (**B**).

Our study group included 21 patients suffering from asthma, 9 of whom showed receptor expression in the polyp tissue. Patients with asthma had significantly higher receptor expression in the polyp tissue (*p* = 0.030) but not in the inferior turbinate tissue (*p* = 0.077) (Figure 5).

The age of the diagnosis, BMI, Lund–Kennedy score, the number of previous surgeries, PTC tasting, H-1 blockers usage, topical and systemic steroids usage, and history of allergies did not show any correlation with the *TAS2R38* receptor expression (*p* > 0.05).

## 3. Discussion

The expression of *TAS2R38* bitter teste receptor in sinonasal respiratory epithelial cells and its functionality was first confirmed in 2012 by Lee et al. [33]. Since then, there have been many studies on its importance in the course and treatment of chronic rhinosinusitis. Thus far, most researchers have focused on studying the expression of the *TAS2R38* receptor in sinonasal epithelial tissue [8,9,10,33,39,44]. Material was collected from various places, such as the ostiomeatal complex, olfactory cleft area, or middle and inferior turbinate. The expression study methods differed from immunohistochemistry, Western Blot and qPCR. In our study, we collected the epithelial mucosa from the inferior turbinate and the pathological polypoid tissue itself. For the expression measurement technique, we chose qPCR [45]. With this study, to the best of our knowledge, we have confirmed the expression of *TAS2R38* receptors in nasal polyp tissue for the first time.

Due to the complex causality, unpredictable course, and varied response to treatment of CRS, we are still looking for more precise tools that would allow us to identify patients who are predisposed to a severe disease course and often require multiple surgeries. It seems that recently the importance of the bitter taste receptor’s role in the inflammatory processes of the upper respiratory tract is rising. Our data confirms the presence of *TAS2R38* receptors not only in the sinonasal tissue but also in the pathological polypoid tissue in patients suffering from CRSwNP. We found a significant difference between the expression level of the receptor in the inferior turbinate mucosa and the patient’s subjective symptoms scoring on the VAS scale. We did not, however, confirm a statistically significant difference between the expression levels of the *TAS2R38* receptor in the sinonasal mucosa of the control group and the sinonasal mucosa and polyps in the study group. There was also no correlation between the severity of the disease and the *TAS2R38* expression levels in our study group; however the Lund–Mackay score showed a tendency indicating a greater advancement of the disease in the CT image in patients with a lower level of expression in the inferior turbinate mucosa. Overall, our results suggest that there might be a correlation between the severity of CRSwNP and the level of the expression of *TAS2R38* in the sinonasal mucosa, but a larger study group might be needed to confirm this tendency.

In our study, we found no significant differences in the perception of the bitter taste of PTC between the study and control group. Due to the early discovery of the gene determinant of the bitter taste perception of PTC, this substance became the basic method of studying the perception of this taste [46]. It has also been widely used in research on the relationship between bitter tasting and chronic rhinosinusitis. Rowan et al. in their research indicate that oral PTC-sensing ability may be a convenient marker of increased disease severity in CRS patients without polyps and appears to provide unique phenotypic information [47]. Correlations between the PTC bitter taste sensing and biofilm formation in CRSsNP patients were also found by Adappa et al. [48]. Interestingly, Linn et al. reported that CRS patients demonstrate decreased sensitivity to quinine, which was significant only in the CRSwNP subgroup, whereas there was no difference in PTC tasting in CRSsNP and CRSwNP subgroups [49]. We can therefore assume that appropriate taste tests should be created separately for patients with and without polyps, because, apparently, these groups differ from each other, and the PTC may not be sufficiently unified.

Our study also showed a significant difference in the expression of the *TAS2R38* receptor in polyp tissue in patients with CRSwNP suffering from asthma. A similar difference was reported by Orsmark-Pietras et al. in their research concerning the *TAS2R* expression in mixed-blood leukocyte samples from healthy children and children with severe therapy-resistant asthma [18]. They stated that the expression of most bitter taste receptors analyzed in the study was higher in children with severe asthma compared to the healthy controls. This and the discovery of the bronchodilatory properties of the bitter taste receptors found in airway epithelium opened new possibilities for the search for therapeutic alternatives for diseases such as asthma and chronic obstructive pulmonary disease (COPD) [50]. Our confirmation of the presence of *TAS2R38* receptors in the sinonasal mucosa and the discovery of their presence in the polyp tissue may also be used for further research to expand the therapeutic possibilities for patients with CRSwNP.

Recently, not only have many new substances that activate *TAS2R38* receptors been discovered, but also their activation by polyphenols, some antibiotics, and substances produced by bacteria have been proven [51,52,53,54]. Perhaps in the future we will be able to act directly on the bitter taste receptors in the sinonasal mucosa with these substances, preventing the development of chronic inflammation and possibly even reducing the already existing polypoid lesions by acting directly on polyp itself. Such a possibility could be an alternative to the dynamically developing, but very expensive, biological therapy, used more and more widely also in CRS [55].

The present study, however, is limited by the relatively small sample size, which may be the reason why some associations were not statistically significant. We believe that the research on bitter taste receptors should be continued, due to the possibility of their potential use both in the treatment and qualification of patients who require more careful monitoring. Another limitation is the evaluation of expression at the mRNA level, which may not fully reflect the actual protein expression, although more and more studies confirm comparable results for both approaches or indicate the advantage of the approach using more standardized techniques [56,57,58].

## 4. Materials and Methods

### 4.1. Patients’ Enrollment and Clinical Data Collection

The research included 146 patients. All patients signed voluntary, informed consent to the biopsies and further genetic tests. The study was approved by the Bioethics Committee of the Medical University of Lodz (RNN/05/18/KE).

The study group inclusion criteria involved any patient 18 years old or older of European descent undergoing functional endoscopic sinus surgery (FESS) for CRSwNP performed in the Department of Otolaryngology, Head and Neck Oncology, Medical University of Lodz, The Norbert Barlicki Memorial Teaching Hospital, Lodz, Poland. The control group consisted of any patient 18 years or older of European descent undergoing septoplasty, conchoplasty, or rhinoplasty in the same department, without a history of chronic or acute rhinosinusitis. Patients with known autoimmune dysfunction, immune deficiency, primary ciliary dyskinesia, cystic fibrosis, any history of radiation exposure to the paranasal sinuses, and oncological treatment were excluded from both groups.

In the study group, mucosal tissue was biopsied from the inferior turbinate mucosa and polyps removed from maxillary, ethmoid, or sphenoid sinus during FESS and placed in RNAlater stabilization solution. In the control group, mucosal tissue was biopsied from the inferior turbinate mucosa during septoplasty, conchotomy, or rhinoplasty and placed in RNAlater stabilization solution. The material was collected by an experienced rhinosurgeon and stored in a freezer at −20 °C until transportation to the laboratory and at −80 °C until nucleic acid isolation.

All patients were tested for PTC (phenylthiocarbamide) tasting ability by administering 2 drops of PTC 0.025% aqueous solution to the tongue (Kolchem, Lodz, Poland). As a positive result, we defined the patient’s perception of bitter taste after administration of drops, and negative results as no bitter taste sensation.

Patients’ metrics, such as age, height, weight, patient’s age at the time of first symptoms, the severity of clinical symptoms according to the Visual Analog Scale (VAS) and Sino-nasal Outcome Test-22 (SNOT-22), additional information about asthma, chronic obstructive pulmonary disease (COPD), allergy, medication applied, and hypersensitivity to non-steroidal anti-inflammatory drugs (NSAIDs), were obtained through a questionnaire. Visual analog scale (VAS) scale was presented to the patients from 0 to 10, where “0” indicated no symptom presence and “10” signified the most severe nasal blockage/obstruction/congestion or nasal discharge (anterior/posterior nasal drip), facial pain/pressure and reduction or loss of smell. The score was evaluated before surgery. Mild symptoms were defined as a VAS score of 0–3 inclusive, moderate as >3–7 inclusive, and severe as ≥7, based on the results of a validation study [59]. The SNOT-22 test was completed by each patient by answering all 22 questions based on a 0–5 scale, where 0 defines no problems with the given symptom and 5 defines maximal problems [60]. 

The severity of the disease in endoscopic nasal examination and in the computed tomography of the paranasal sinuses was analyzed according to Lund–Mackay and Lund–Kennedy scales. Endoscopic examination of the nasal cavities was graded in a 3-point classification system (0—absence of polyps; 1—polyps in middle meatus only; 2—polyps beyond middle meatus but not blocking the nose completely; 3—polyps completely obstructing the nose) [61]. The Lund–Mackay system was used to stage the computed tomography (CT) scans conducted before the surgery [33]. Scoring: for all sinus systems, except the ostiomeatal complex: 0 = no abnormalities, 1 = partial opacification, 2 = total opacification. For the ostiomeatal complex: 0 = not occluded, 2 = occluded. A total score of 0 to 24 is possible, and each side can be considered separately (0 to 12). A mild disease was defined as a CT score of 0–7 inclusive, moderate from 8–15, and severe from 16–24.

### 4.2. RNA Isolation and TAS2R38 Expression Analysis

Total RNA was isolated from nasal mucosa biopsies using Maxwell^®^ RSC simplyRNA Tissue Kit (Promega, Madison, WI, USA; cat No AS1340). The RNA was DNAse treated with TURBO DNA-*free*^TM^ Kit (Ambion, Thermofisher, Waltham, MA, USA; cat No AM1907). The concentration and purity of the RNA were measured on Nanodrop spectrophotometer (Thermo Scientific; Thermofisher, Waltham, MA, USA). Complementary DNA was generated using 500 ng of total RNA and High-capacity cDNA Reverse Transcription Kit (Applied Biosystem, Waltham, MA, USA Cat No 4374966), according to the manufacturer’s instructions on Thermal Cycler, Bio-Rad T-100 (BioRad Laboratories Inc., Hercules, CA, USA, cat No 1861096) [62,63]. 

Real-time polymerase chain reaction (qRT-PCR) was performed in duplicates using TaqMan^TM^ Fast Advanced Master Mix (Applied Biosystem, Thermofisher, Waltham, MA, USA; cat No 4444556) and TaqMan^TM^ Gene expression assays for the *TAS2R38* gene (Applied Biosystem Hs00604294_s1, Thermofisher, Waltham, MA, USA; cat No 4331182) and for reference genes: *18S RNA* (Applied Biosystem Hs03928990_g1, Thermofisher, Waltham, MA, USA; cat No 4331182), *β-actin* (ACTB, Applied Biosystem Hs4333762T_g1, Thermofisher, Waltham, MA, USA; cat No 4331182) and *GAPDH* (Applied Biosystem, Hs02786624_g1, Thermofisher, Waltham, MA, USA; cat No 4331182) on Bio-Rad CFX 96 (BioRad Laboratories Inc., Hercules, CA, USA) [64].

### 4.3. Reference Gene Selction

Glyceraldehyde 3-phosphate dehydrogenase gene (*GAPDH*) was the most used previously in studies on bitter taste receptors expression with qRT-PCR [41,42,43,44]. The other reference gene widely studied was 18S ribosomal RNA *(18S RNA)*, but most often it was collated with another gene such as *β-actin* or, as already mentioned, *GAPDH* [17,21]. Only in a few publications were the results normalized only to *β-actin* [12,65]. To select the most suitable gene for our research, we decided to evaluate the expression of all three genes and calculate the coefficient of variation (CoV) of CT values for each gene exclusively for polyp tissue, exclusively for inferior turbinate mucosa (ITM), and for both for polyp and ITM tissues. Additionally, a Wilcoxon test was performed in order to investigate the difference in medians between polyp and ITM CT values for each gene candidate, and the number of samples with non-zero expression of potential normalizer was evaluated.

### 4.4. Statistical Analysis

Statistical analyses were conducted in Statistica TIBCO version 13.0 and MS Excel. Continuous variables are presented as arithmetical means with standard deviations or medians with the values of the lower and upper quartile, depending on the normality of the distribution evaluated using the Shapiro–Wilk test. Nominal variables are presented as numbers with corresponding percentages. For quantitative variables, differences between groups were evaluated using the Mann–Whitney U test. Correlation coefficients were calculated using the Spearman rank method. For nominal variables, Pearson’s χ2 with Yates correction or Fisher’s exact test was performed, depending on the size of the group. For paired observations, McNemar’s test was performed. *p* values < 0.05 were considered statistically significant [66,67].

## 5. Conclusions

Chronic rhinosinusitis with nasal polyps is one of the diseases that significantly reduce the quality of life of patients. Its complex causality and hard-to-predict course force a constant search for new diagnostic possibilities and alternative methods of treatment. Bitter taste receptors have now become one of the fields of this research, due to their wide presence in the upper and lower respiratory tract and proven immune defense mechanisms. Here, we show that PTC, even though is a widely used substance in the bitter taste research, may not be sufficient in CRS patient studies. We proved the presence of the *TAS2R38* receptor not only in the ITM of the control group but also in ITM as well as in polypoid tissue of CRSwNP patients. We also report differences indicating a reduced expression of the *TAS2R38* receptor in the ITM in patients with more severe subjective symptoms and more clinically advanced lesions.

## Figures and Tables

**Table 1:** Characteristics of the study (N = 107) and the control (N = 39) groups. F = female, M = male, BMI = body mass index, PTC = phenylthiocarbamide, SNOT-22 = Sino-nasal Outcome Test-22, VAS = visual analog scale, SD—standard deviation, IQR—interquartile range.

		Control Group (N = 39)	Study Group (N = 107)
Gender	F	14 (35.90%)	44 (51.12%)
	M	25 (64.10%)	63 (58.88%)
Mean Age (SD)		37.90 (12.29)	46.27 (13.74)
Weight		78.92 (11.92)	80.59 (16.52)
Height		172 (9.81)	173 (9.23)
BMI		26.68 (4.46)	26.89 (4.61)
PTC tasting	0	13 (33,33%)	35 (32.71%)
	1	26 (66.67%)	72 (67.29%)
H-1 blockers	0	37 (94.87%)	66 (61.68%)
	1	2 (5.13%)	41 (38.32%)
Topical steroids	0	36 (92.31%)	44 (41.12%)
	1	3 (7.69%)	63 (58.88%)
Systemic steroids	0	39 (100.00%)	101 (94.39%)
	1	0 (0.00%)	6 (5.61%)
Seasonal allergies	0	38 (97.44%)	84 (78.50%)
	1	1 (2.56%)	23 (21.50%)
Year-round allergies	0	36 (92.31%)	78 (72.90%)
	1	3 (7.69%)	29 (27.10%)
Tobacco usage		12 (30.77%)	16 (14.95%)
Median SNOT-22 score (IQR)		32 (27–37)	
VAS score	0–3		55 (51.40%)
	4–7		29 (27.10%)
	8–10		23 (21.50%)
Lund Kennedy score	0–2		59 (55.14%)
	3–4		45 (42.06%)
	5–6		3 (2.80%)
Median Lund-Mackay score (IQR)			9 (6–14)

**Table 2:** Coefficients of variations (CoV) calculated for inferior turbinate mucosa (ITM), polyp, and all samples. Assessed differences for cycle thresholds between polyp and inferior turbinate mucosa samples are presented as Wilcoxon *p* values.

	Count of Non-Zero Expression Samples	CoV ITM	CoV Polyp	CoV Total	Wilcoxon *p*
*β-actin*	219	10.96%	19.62%	16.14%	0.092
GAPDH	190	9.15%	10.92%	10.07%	0.335
18S	226	21.84%	27.01%	28.30%	0.003

**Table 3:** Median (25–75%) of *TAS2R38* receptor expression levels normalized to *β-actin* in polypoid tissue and inferior turbinate tissue.

	Control Group (N = 28)	Study Group (N = 43)
*Inferior turbinate mucosa*	0.000123 (0.00042–0.00078)	0.00013 (0.00006–0.00028)
*Polyp*	X	0.00017 (0.00004–0.00078)

## Data Availability

Data available upon request.

## Abbreviations

CRS	Chronic rhinosinusitis
CRSwNP	Chronic rhinosinusitis with nasal polyps
CRSsNP	Chronic rhinosinusitis without nasal polyps
qRT-PCR	Real-time polymerase chain reaction
PDAC	Human pancreatic ductal adenocarcinoma
SNPs	Single nucleotide polymorphisms
NO	Nitric oxide
NOS	Nitric oxide synthase
AHLs	Acyl-homoserine lactones
C4HSL	N-butyryl-L-homoserine lactone
C12HSL	N-3-oxo-dodecanoyl-L-homoserine lactone
Ca2+	Calcium ion
K+	Potassium ion
PLCβ2	Phospholipase C isoform β2
IP3	Inositol trisphosphate
PTC	Phenylthiocarbamide
SNOT-22	Sino-nasal Outcome Test-22
VAS	Visual Analog Scale
F	Female
M	Male
BMI	Body mass index
IQR	Interquartile range
COPD	Chronic obstructive pulmonary disease (COPD
FESS	Functional endoscopic sinus surgery
NSAIDs	Non-steroidal anti-inflammatory drugs
CT	Computed tomography
18S RNA	18S ribosomal RNA
ACTB	β-actin
GAPDH	Glyceraldehyde 3-phosphate dehydrogenase gene
CoV	Coefficient of variation
ITM	Inferior turbinate mucosa
